# Elucidation of the mechanisms underlying tumor aggravation by the activation of stress-related neurons in the paraventricular nucleus of the hypothalamus

**DOI:** 10.1186/s13041-023-01006-0

**Published:** 2023-02-02

**Authors:** Sara Yoshida, Yusuke Hamada, Michiko Narita, Daisuke Sato, Kenichi Tanaka, Tomohisa Mori, Hiroyuki Tezuka, Yukari Suda, Hideki Tamura, Kazunori Aoki, Naoko Kuzumaki, Minoru Narita

**Affiliations:** 1 Department of Pharmacology, Hoshi University School of Pharmacy and Pharmaceutical Sciences, 2-4-41 Ebara, Shinagawa-Ku, Tokyo, 142-8501 Japan; 2 Division of Cancer Pathophysiology, National Cancer Center Research Institute (NCCRI), 5-1-1 Tsukiji, Chuo-Ku, Tokyo, 104-0045 Japan; 3 Department of Cellular Function Analysis, Research Promotion Headquarters, Fujita Health University, 1-98 Dengakugakubo, Kutsukake-Cho, Toyoake, Aichi 470-1192 Japan; 4 Institute for Advanced Life Sciences, Hoshi University School of Pharmacy and Pharmaceutical Sciences, 2-4-41 Ebara, Shinagawa-Ku, Tokyo, 142-8501 Japan; 5 Laboratory of Biofunctional Science, Hoshi University School of Pharmacy and Pharmaceutical Sciences, 2-4-41 Ebara, Shinagawa-Ku, Tokyo, 142-8501 Japan; 6 Department of Immune Medicine, National Cancer Center Research Institute (NCCRI), 5-1-1 Tsukiji, Chuo-Ku, Tokyo, 104-0045 Japan

**Keywords:** Corticotropin-releasing hormone, Cancer, Tumor-infiltrating lymphocytes, Tumor-associated macrophage, Glucocorticoids, Tsc22d3

## Abstract

A growing body of evidence suggests that excess stress could aggravate tumor progression. The paraventricular nucleus (PVN) of the hypothalamus plays an important role in the adaptation to stress because the hypothalamic–pituitary–adrenal (HPA) axis can be activated by inducing the release of corticotropin-releasing hormone (CRH) from the PVN. In this study, we used pharmacogenetic techniques to investigate whether concomitant activation of CRH^PVN^ neurons could directly contribute to tumor progression. Tumor growth was significantly promoted by repeated activation of CRH^PVN^ neurons, which was followed by an increase in the plasma levels of corticosterone. Consistent with these results, chronic administration of glucocorticoids induced tumor progression. Under the concomitant activation of CRH^PVN^ neurons, the number of cytotoxic CD8^+^ T cells in the tumor microenvironment was dramatically decreased, and the mRNA expression levels of hypoxia inducible factor 1 subunit α (HIF1α), glucocorticoid receptor (GR) and Tsc22d3 were upregulated in inhibitory lymphocytes, tumor-associated macrophages (TAMs) and myeloid-derived suppressor cells (MDSCs). Furthermore, the mRNA levels of various kinds of driver molecules related to tumor progression and tumor metastasis were prominently elevated in cancer cells by concomitant activation of CRH^PVN^ neurons. These findings suggest that repeated activation of the PVN-CRHergic system may aggravate tumor growth through a central–peripheral-associated tumor immune system.

**Supplementary Information:**

The online version contains supplementary material available at 10.1186/s13041-023-01006-0.

## Introduction

Stress has been defined as “the non-specific response of the body to any demand for change,” and is classified into two types based on Selye’s theory [1]. While “eustress” is positive stress that has beneficial effects on the body, “distress” is linked to stressors and has deleterious effects on the body by exceeding the ability of the individual to cope. In general, it is believed that distress can trigger both psychiatric and physical disorders.

Stress affects several brain areas including limbic areas, such as the hippocampus, amygdala and prefrontal cortex. Accumulating evidence suggests that stress promotes the aggravation of anxiety, schizophrenia, cognitive disorders and depression [2–7]. Furthermore, the paraventricular nucleus (PVN) of the hypothalamus has been shown to play a critical role in the adaptation to stress. Neurons in the PVN that contain corticotropin-releasing hormone (CRH) (CRH^PVN^) play a particularly important role in the response to stress. In addition, the hypothalamic–pituitary–adrenal (HPA) axis is a major descending stress-response pathway from the hypothalamus that regulates various physiological responses [8].

Cancer, where cells proliferate uncontrollably, can be regulated by the microenvironment surrounding tumor cells, like angiogenesis, immune cells, fibroblasts and cytokines. We previously demonstrated that concomitant activation of D1-receptor-positive medium spiny neurons in the nucleus accumbens, which is a brain region that is closely related to motivation and stress, suppressed tumor progression while improving the immune system [9], indicating that positive effects on motivation may negatively influence tumor progression. However, little is known about the dynamic interaction between the stress-related, especially CRH^PVN^ neuron-mediated, neural response and cancer development. Therefore, we hypothesized that CRH^PVN^ neurons could affect the progression of cancer. In the present study, by using designer receptors exclusively activated by designer drugs (DREADD) systems, we investigated whether a change in the activity of CRH^PVN^ neurons could directly contribute to tumor progression mediated through the central–peripheral-associated immune system.

## Materials and methods

### Animals

C57BL/6J mice (Tokyo Laboratory Animals Science, Tokyo, Japan) and CRH-ires-Cre mice [B6(Cg)-*Crh*^*tm1(cre)Zjh*^/J; Stock #012704, Jackson Laboratory, Bar Harbor, ME, USA] were used for this study. All mice were housed individually and maintained under a room temperature of 24 ± 1 °C with a 12-h light–dark cycle (lights on 8:00 a.m. to 8:00 p.m.). Food and water were available ad libitum during every experimental period. All experiments were conducted in accordance with the ethical guidelines for the care and use of laboratory animals of Hoshi University School of Pharmacy and Pharmaceutical Sciences.

### Stereotaxic AAV injection

Mice were anesthetized with isoflurane (3%, inhalation) before surgery. At the start of the surgical procedures, mice were fixed in a stereotaxic apparatus (RWD Life Science, CA, USA). For manipulation of CRH neurons in the PVN, AAV10-hSyn-Flex-hM3Dq-mCherry was delivered bilaterally to the PVN of male CRH-ires-Cre mice (A/P, − 0.69 mm; M/L, ± 1.06 mm from the bregma; D/V, − 4.66 mm from the dura; with a 10° angle toward the midline in the coronal plane) through an internal cannula (Eicom Co., Kyoto, Japan) at a rate of 0.25 μL/min for 4 min (1 μL total volume per side) with a glass micropipette and an air pressure injector system (Micro-syringe Pump-Model ESP-32; Eicom Co.). After each injection, the cannula was left in the brain for 2 min. After the surgical procedures, mice were allowed to recover in their home cages for at least 2 weeks until AAV vector-derived transgene expressed enough Gq-coupled human muscarinic M3 (hM3Dq). The virus used in this study was a kind gift from Dr. Akihiro Yamanaka (Nagoya University, Nagoya, Japan).

### Drug treatment

Hydrocortisone (10 mg/kg, FUJIFILM Wako Pure Chemical Corp., Osaka, Japan), suspended in physiological saline (Otsuka Pharmaceutical Factory Inc., Tokushima, Japan) containing 5% (v/v) Tween-80 (Sigma-Aldrich, Inc., MO, USA), was injected intraperitoneally once a day for 20 days. The dose (10 mg/kg) of hydrocortisone used in the present study was chosen based on the results of a previous study using rodents [10]. Clozapine *N*-oxide (CNO; 3 mg/kg, Abcam plc., Cambridge, UK) to activate hM3Dq was dissolved in saline.

### Electrophysiological evaluation of hM3Dq receptor activation by CNO

Whole-cell recordings from PVN neurons were performed as previously described [9]. Briefly, coronal slices (250 µm thick) were cut on a vibratome (VT-1200S, Leica Biosystems, Wetzlar, Germany) in oxygenated ice-cold sucrose-based solution. Slices were transferred to an oxygenated artificial cerebrospinal fluid (ACSF) containing (in mM): 128 NaCl, 3 KCl, 2 MgCl_2_, 2 CaCl_2_, 24 NaHCO_3_, 1.25 NaH_2_PO_4_, and 10 glucose. The slices were kept in ACSF to recover for at least 1 h before recording. Patch recording electrodes (4–6 MΩ) were filled with K-gluconate solution containing biocytin. CRH neurons expressing hM3Dq-mCherry in the PVN were visually identified with a Nikon FN-1 fluorescence microscope (Nikon, Tokyo, Japan). Data were acquired using a Multiclamp 700B amplifier (Molecular Devices, Sunnyvale, CA, USA) and analyzed using pCLAMP 10 software (Molecular Devices). In the current-clamp mode, after a stable membrane potential was recorded, CNO was bath-applied to determine the effects of hM3Dq receptor activation. After recording, slices were fixed in 4% paraformaldehyde, and biocytin-filled neurons were visualized using streptoavidin-conjugated Alexa Fluor-350 (1:2000; S11249, Thermo Fisher Scientific Inc., Waltham, MA, USA).

### Cancer cell implantation

Lewis lung carcinoma (LLC) cells and LLC-luc cells were suspended with extracellular matrix gel (Sigma-Aldrich, Inc.) and Hank’s Balanced Salt solution (Thermo Fisher Scientific Inc.) at a concentration of 1 × 10^6^ cells/mL. The cell suspension (1 × 10^6^ cells/mL; 0.5 mL) was transplanted subcutaneously into the right lower back of WT/hM3Dq mice, CRH-Cre/hM3Dq mice and C57BL/6J mice that had been injected with vehicle or hydrocortisone.

### Graft tumor growth assay

After tumor implantation, tumor size was measured using calipers, and tumor volume was calculated as (L × W^2^)/2, (L = length and W = width). In CRH-Cre/hM3Dq mice, WT/hM3Dq mice and C57BL/6J mice that had been injected with vehicle or hydrocortisone, tumor size was measured every other day for 18–21 days after implantation.

### Corticosterone ELISA

Blood was collected from the abdominal aorta of mice by a 26 gauge needle (TERUMO Corp., Osaka, Japan), and transferred to a collection tube that contained an anticoagulant such as EDTA under isoflurane anesthesia (3%, inhalation). To separate the plasma fraction, the blood sample was centrifuged at 5000 rpm for 5 min at room temperature, and the upper layer was then gently collected. The assay was performed using the DetectX® Corticosterone Enzyme Immunoassay Kit (Arbor Assays, Ann Arbor, MI, USA) according to the instructions from the manufacturer.

### Flow cytometry

Under isoflurane anesthesia (3%, inhalation), spleen was harvested from the mice for the analysis of spleen-derived immune cells, and then homogenized using a syringe plunger with PBS (Thermo Fisher Scientific Inc.). After the cell suspension was filtered through a nylon mesh and 100 μm cell strainer to remove cell aggregates, it was then treated with ammonium chloride hemolysis.

For the analysis of tumor-infiltrating lymphocytes (TILs) and LLC-luc cells, tumor tissue (< 1.0 g) was harvested from the mice under isoflurane anesthesia (3%, inhalation) and minced into small pieces. 2× Tumor & Tissue Dissociation Reagent (TTDR; Becton, Dickinson and Company, Franklin Lakes, NJ, USA) diluted with Dulbecco’s Modified Eagle Medium (DMEM; Thermo Fisher Scientific Inc.) was added to the tissue suspension, and the mixture was then incubated for 30 min at 37 °C. After the incubation period, 1% Albumin, from Bovine Serum, Fraction V pH7.0 (BSA; FUJIFILM Wako Pure Chemical Corp.)/2 mM ethylenediaminetetraacetic acid (EDTA; NIPPON GENE Co., Ltd., Tokyo, Japan) in PBS (25 mL) was added to the suspension. The homogenized suspension was segregated using a 70 μm cell strainer to remove cell aggregates. Subsequently, a hemolytic agent ammonium chloride was added to a cell suspension.

Before specific extracellular staining to classify immune cells, cells were incubated with an anti-CD16/32 antibody (Becton, Dickinson and Company) to avoid non-specific binding. Next, cells were stained with the following antibodies; APC/Cy-conjugated anti-CD45.2 (104), FITC or PE-conjugated anti-CD4 (GK1.5), PE-conjugated anti-CD8a (53–6.7), PE or APC-conjugated anti-CD3ε (145-2C11), APC-conjugated anti-CD49b (DX5), PE/Cy7-conjugated anti-Ly6G (1A8), APC-conjugated anti-CD11b (M1/70), PE-conjugated anti-Ly6C (HK1.4), and PE-conjugated anti-F4/80 (BM8). All antibodies were purchased from BioLegend (San Diego, CA, USA). Dead cells were segregated by staining with propidium iodide (PI; Sigma-Aldrich, Inc.) or 7-aminoactinomycin D (7-AAD; Becton, Dickinson and Company). Immune cells were sorted and analyzed using a BD FACS Aria™ II Cell Sorter (Becton, Dickinson and Company) and Flowjo™ software (Becton, Dickinson and Company).

### Quantitative real-time reverse-transcription PCR (qRT-PCR) for mRNA

Total RNA obtained from immune cells of the mice described above was extracted using a mirVana miRNA Isolation Kit (Thermo Fisher Scientific Inc.), and purified total RNA was then reverse-transcribed into cDNA using a SuperScript VILO cDNA Synthesis Kit (Thermo Fisher Scientific Inc.) according to the manufacturer’s protocol. qPCR was performed using a StepOne Plus™ System (Thermo Fisher Scientific Inc.) with a Fast SYBR® Green Master Mix (Thermo Fisher Scientific Inc.) and synthesized primers (Additional file 2: Table S1). Glyceraldehyde-3-phosphate dehydrogenase (GAPDH) was used as a normalization control.

### Statistical analysis

The results are presented as the mean ± S.E.M. The statistical significance of differences between groups was determined using an unpaired *t*-test or two-way ANOVA followed by Bonferroni’s multiple comparisons test. Statistical analyses and graph generations were performed with GraphPad Prism 9.0 (GraphPad Software, San Diego, CA, USA).

## Results

### Chronic activation of CRH^PVN^ neurons promotes tumor growth due to the secretion of excess glucocorticoids

**Fig. 1:**
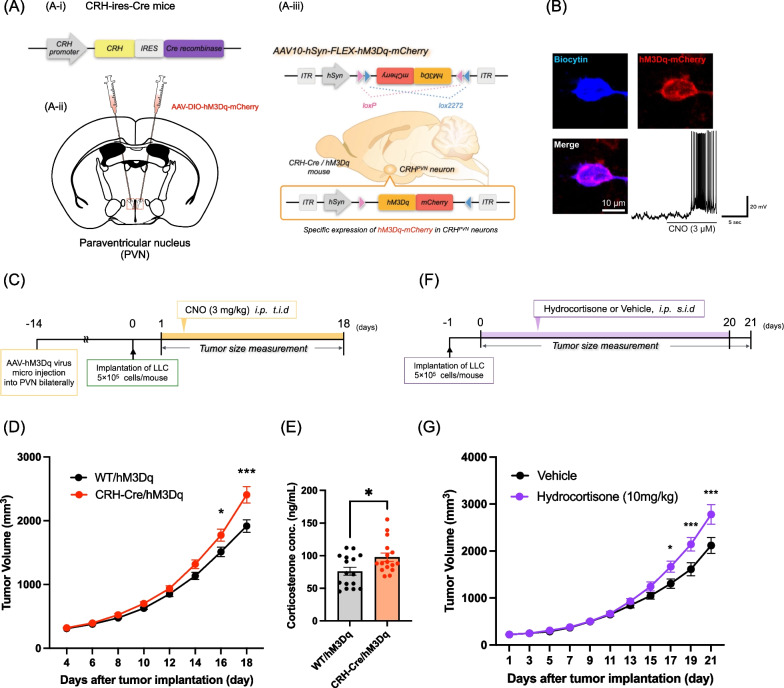
Chronic activation of CRH^PVN^ neurons promotes tumor progression through the excess secretion of glucocorticoids. **A** Schematic images of the specific activation of CRH^PVN^ neurons by the injection of AAV-derived transgenes into the PVN of CRH-ires-Cre mice. Schematic illustrations of CRH-ires-Cre mice (**A-i**), AAV injection site (**A-ii**) and AAV10-hSyn-Flex-hM3Dq-mCherry vector (**A-iii**). **B** Representative images of biocytin-labeled patched neuron and hM3Dq-mCherry in the PVN. Cell with blue (biocytin), red (hM3Dq-mCherry), and purple (merged) fluorescence. Voltage trace recorded from an hM3Dq-mCherry expressing CRH neuron during the application of CNO (3 μM) in a slice including the PVN. **C** Experimental schedule for evaluating tumor growth by the activation of CRH^PVN^ neurons. **D** Quantitative analysis of tumor volume in WT/hM3Dq mice and CRH-Cre/hM3Dq mice. The results are presented as the mean ± S.E.M. The data were subjected to a comparative analysis by two-way ANOVA followed by the Bonferroni test: *p < 0.05, ***p < 0.001 vs. WT/hM3Dq mice (n = 22). **E** Plasma levels of corticosterone as measured by ELISA. Data are presented as the mean ± S.E.M. Unpaired *t*-test: *p < 0.05 vs. WT/hM3Dq mice (n = 16). **F** Experimental schedule for evaluating tumor growth by chronic exposure to hydrocortisone. **G** Quantitative analysis of tumor volume in the vehicle group and hydrocortisone group (10 mg/kg). Data are presented as the mean ± S.E.M. The data were subjected to a comparative analysis by two-way ANOVA followed by the Bonferroni test: *p < 0.05, ***p < 0.001 vs. vehicle group (n = 6)

First, we investigated whether chronic activation of CRH^PVN^ neurons could change tumor growth using the Gq-designer receptors exclusively activated by designer drugs (DREADD) system. To selectively manipulate the activity of CRH^PVN^ neurons, a Cre-dependent AAV carrying a construct of hM3Dq was injected into the PVN of CRH-Cre mice (Fig. 1A). Under these conditions, electrophysiological recording showed that hM3Dq-mCherry-positive neurons in the PVN were depolarized and fired action potentials after bath application of CNO (Fig. 1B). After LLC cell implantation into the right lower back of mice, CNO was administered repeatedly and tumor size was measured with a caliper (Fig. 1C). As a result, tumor growth in CRH-Cre/hM3Dq mice was greater than that in WT/hM3Dq mice (Fig. 1D, two-way ANOVA followed by the post-hoc Bonferroni test, *p < 0.05, ***p < 0.001 vs. WT/hM3Dq). We also measured the plasma levels of corticosterone in these mice. By repeated activation of CRH^PVN^ neurons, the plasma levels of corticosterone in CRH-Cre/hM3Dq mice were significantly greater than those in WT/hM3Dq mice (Fig. 1E, Unpaired *t*-test, *p < 0.05 vs. WT/hM3Dq). Based on these results, we hypothesized that chronic exposure to glucocorticoids could promote tumor growth. To investigate the effect of glucocorticoids on tumor growth, hydrocortisone was injected repeatedly after LLC implantation (Fig. 1F). As a result, tumor volume was dramatically increased under chronic exposure to hydrocortisone compared to that in the vehicle group (Fig. 1G, two-way ANOVA followed by the post-hoc Bonferroni test, *p < 0.05, ***p < 0.001 vs. vehicle group). Taken together, these results suggest that repeated activation of CRH^PVN^ neurons can promote tumor growth through excess secretion of glucocorticoids.

### Decrease in cytotoxic tumor-infiltrating lymphocytes through the activation of CRH^PVN^ neurons

To investigate the mechanisms of tumor progression by the activation of CRH^PVN^ neurons, we focused on immune systems. First, we analyzed several immune cells derived from the spleen of these mice using flow cytometry. The numbers of various kinds of immune cells were not changed in the spleen of CRH-Cre/hM3Dq mice (Additional file 1: Fig. S1A, B), whereas among cytotoxic protein genes, granzyme B, perforin and interferon-γ (IFN-γ), in splenic NK cells, the mRNA level of perforin in CRH-Cre/hM3Dq mice was significantly less than that in WT/hM3Dq mice (Additional file 1: Fig. S1C, Unpaired *t*-test, *p < 0.05 vs. WT/hM3Dq).

**Fig. 2:**
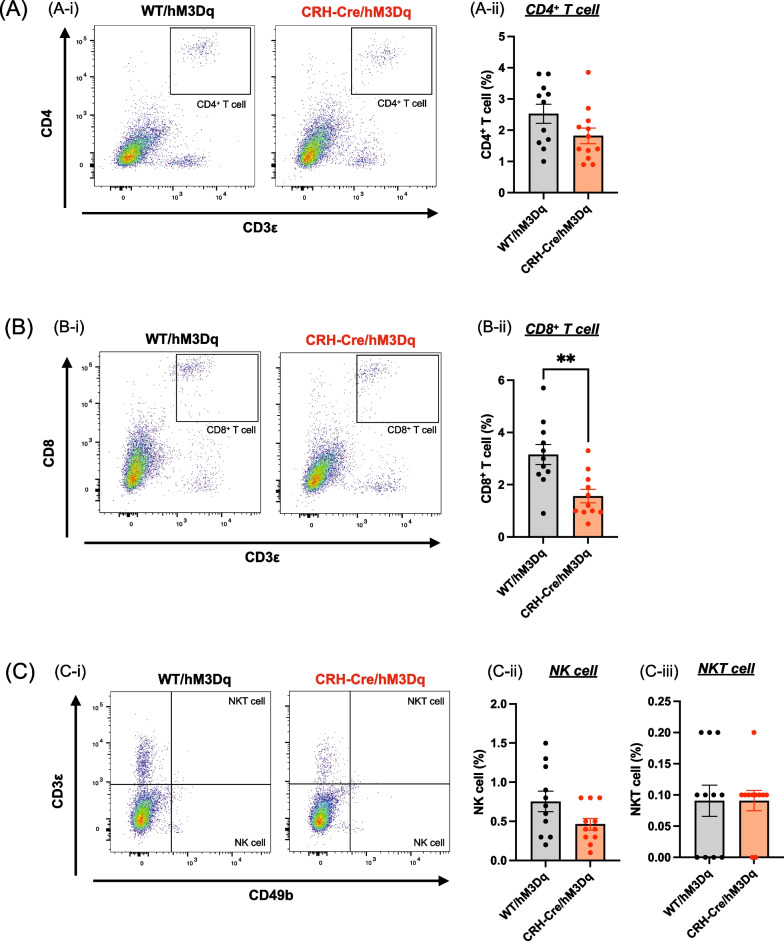
Effects of the activation of CRH^PVN^ neurons on cytotoxic tumor-infiltrating lymphocytes. **A**–**C** Representative flow cytometric dot plots (left panel) and quantitative analyses of CD4^+^ T cells (**A**), CD8^+^ T cells (**B**), NK cells and NKT cells (**C**) derived from tumor-infiltrating lymphocytes (TILs) of WT/hM3Dq or CRH-Cre/hM3Dq mice. Each point represents the mean ± S.E.M. Unpaired *t*-test: **p < 0.01 vs. WT/hM3Dq mice (n = 11–12)

In TILs, the population of CD4^+^ T cells tended to be decreased, whereas the population of NK cells and NKT cells was not changed in CRH-Cre/hM3Dq mice compared to that in WT/hM3Dq mice (Fig. 2A, C). However, the number of CD8^+^ T cells was significantly decreased in CRH-Cre/hM3Dq mice compared to that in WT/hM3Dq mice, indicating that infiltrating cytotoxic lymphocytes were significantly decreased by repeated activation of CRH^PVN^ neurons (Fig. 2B, Unpaired *t*-test, **p < 0.01 vs. WT/hM3Dq).

### Changes in tumor-associated macrophages (TAMs) and myeloid-derived suppressor cells (MDSCs) under the chronic activation of CRH^PVN^ neurons

**Fig. 3:**
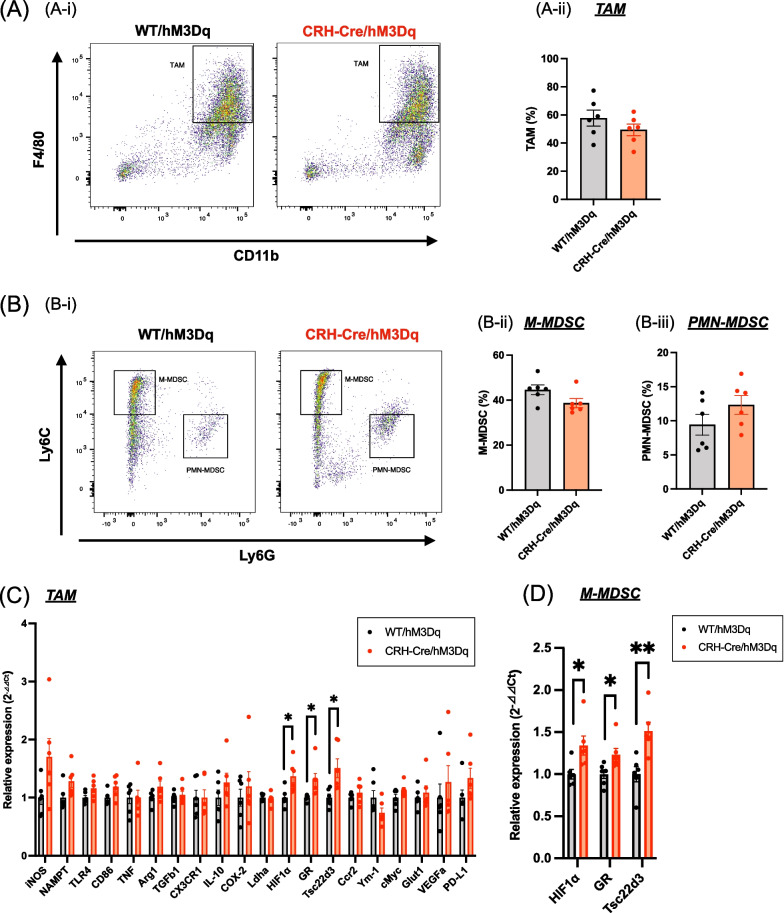
Effects of the activation of CRH^PVN^ neurons on tumor-associated macrophages (TAMs) and myeloid-derived suppressor cells (MDSCs) in tumor microenvironments. **A**, **B** Representative flow cytometric dot plots (left panel) and quantitative analyses of TAM (**A**), M-MDSC and PMN-MDSC (**B**) derived from WT/hM3Dq or CRH-Cre/hM3Dq mice. Each point represents the mean ± S.E.M. (n = 6). **C**, **D** Quantitative PCR analysis of the expression of various mRNAs in TAM (**C**) and M-MDSC (**D**) derived from WT/hM3Dq or CRH-Cre/hM3Dq mice. Each point represents the mean ± S.E.M. Unpaired *t*-test: *p < 0.05, **p < 0.01 vs. WT/hM3Dq mice (n = 6)

Next, we focused on inhibitory immune cells such as TAM and MDSC under these conditions. There were no differences in the populations of TAM or MDSC in the TILs between CRH-Cre/hM3Dq mice and WT/hM3Dq mice (Fig. 3A, B). Next, we investigated changes in the expression of various genes related to pro-tumor functions. As a result, mRNA levels of hypoxia inducible factor 1 subunit α (HIF1α), glucocorticoid receptor (GR) and Tsc22d3 were significantly increased in TAMs derived from CRH-Cre/hM3Dq mice compared to those from WT/hM3Dq mice (Fig. 3C, Unpaired *t*-test, *p < 0.05 vs. WT/hM3Dq). Interestingly, these mRNA levels were also dramatically increased in monocytic MDSCs (M-MDSCs) derived from CRH-Cre/hM3Dq mice compared to those from WT/hM3Dq mice (Fig. 3D, Unpaired *t*-test, *p < 0.05, **p < 0.01 vs. WT/hM3Dq).

### Functional changes in cancer cells under the chronic activation of CRH^PVN^ neurons

**Fig. 4:**
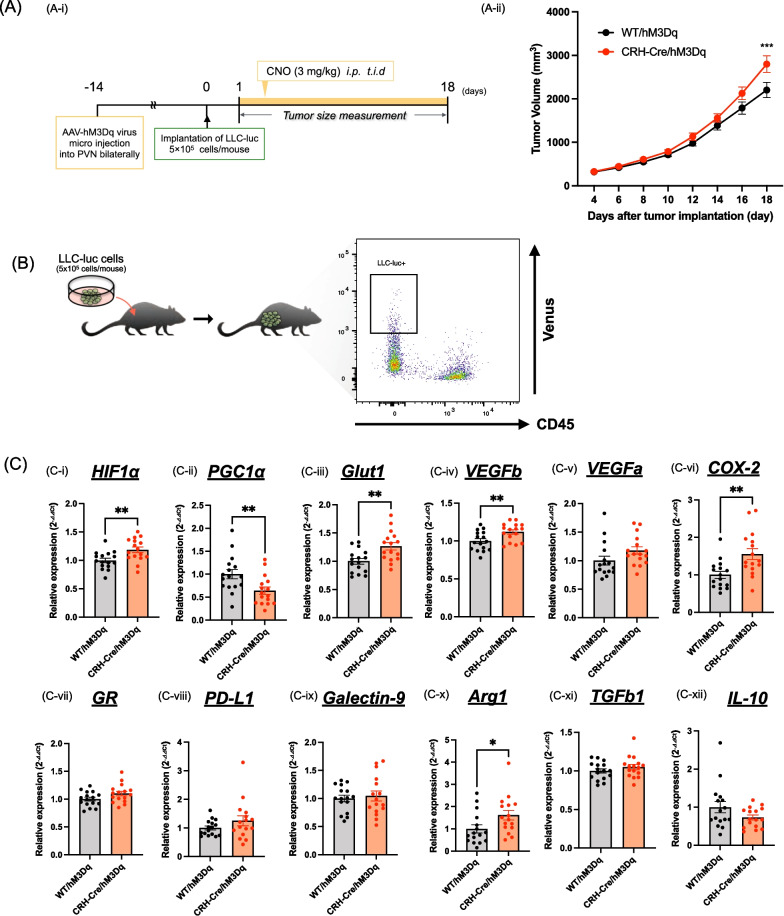
Gene expression analysis of cancer cells derived from tumor tissue under the activation of CRH^PVN^ neurons. **A** Experimental schedule for evaluating gene expression in cancer cells derived from tumor tissue under the activation of CRH^PVN^ neurons (**A-i**). Quantitative analysis of tumor volume in WT/hM3Dq mice and CRH-Cre/hM3Dq mice. All data are presented as the mean ± S.E.M. The data were subjected to a comparative analysis by two-way ANOVA followed by the Bonferroni test: ***p < 0.001 vs. WT/hM3Dq mice (n = 16, **A-ii**). **B** Schematic images of the tumor dissociation strategy. The panel on the right is a representative flow cytometric plot. **C** Quantitative PCR analysis for the expression of various mRNAs in cancer cells derived from tumor tissue. HIF1α (**C-i**), PGC1α (**C-ii**), Glut1 (**C-iii**), VEGFb (**C-iv**), VEGFa (**C-v**), COX-2 (**C-vi**), GR (**C-vii**), PD-L1 (**C-viii**), Galectin-9 (**C-ix**), Arg1 (**C-x**), TGFb1 (**C-xi**) and IL-10 (**C-xii**). Each point represents the mean ± S.E.M. Unpaired *t*-test: *p < 0.05, **p < 0.01 vs. WT/hM3Dq mice (n = 16)

Finally, we investigated whether repeated activation of CRH^PVN^ neurons could modulate the worsening of cancer cells. To evaluate cancer cells, LLC cells that encoded fluorescent protein-fused luciferase (LLC-luc), which could be detected by green fluorescent protein because ff-Luc was fused to the Venus gene, was implanted and then CNO was repeatedly injected to activate CRH^PVN^ neurons (Fig. 4A-i). As shown in Fig. 1D, the growth of LLC-luc was significantly promoted by repeated activation of CRH^PVN^ neurons (Fig. 4A-ii, two-way ANOVA followed by the post-hoc Bonferroni test, ***p < 0.001 vs. WT/hM3Dq). Under these conditions, tumor tissue was harvested from these mice and LLC-luc cells expressing the Venus protein were selected by flow cytometry (Fig. 4B). In these sorted cells, various kinds of gene expression were analyzed by qPCR (Fig. 4C). As a result, the mRNA levels of inhibitory ligands, such as programmed cell death 1 ligand 1 (PD-L1) and galectin-9 in CRH-Cre/hM3Dq mice were not changed (Fig. 4C-viii, ix). The mRNA levels of vascular endothelial growth factor a (VEGFa), glucocorticoid receptor (GR), transforming growth factor-β1 (TGFb1) and interleukin-10 (IL-10) were not changed in these mice (Fig. 4C-v, vii, xi, xii). However, the mRNA levels of glycolysis-related genes like HIF1α, glucose transporter 1 (Glut1), VEGFb, cyclooxygenase-2 (COX-2) and arginase 1 (Arg1) in CRH-Cre/hM3Dq mice were significantly greater than those in WT/hM3Dq mice (Fig. 4C-i, iii, iv, vi, x, Unpaired *t*-test, *p < 0.05, **p < 0.01 vs. WT/hM3Dq). Furthermore, the mRNA level of peroxisome proliferator-activated receptor gamma coactivator1-α (PGC1α) in CRH-Cre/hM3Dq mice was significantly less than that in WT/hM3Dq mice (Fig. 4C-ii, Unpaired *t*-test, **p < 0.01 vs. WT/hM3Dq).

## Discussion

Acute stress stimuli have been shown to facilitate the activation of CRH^PVN^ neurons followed by the HPA axis to maintain homeostasis for the whole body [8]. In contrast, it has been reported that chronic activation of these neurons induces an abnormality of the HPA axis, leading to immune dysfunction as well as various psychiatric disorders accompanied by brain dysfunction [11, 12]. Furthermore, a growing body of evidence suggests that excess stress could affect cancer progression through dysfunction of the autonomic nervous system [13–16]. The key finding of this study was that the concomitant activation of hypothalamic CRHergic neurons by the DREADD system promoted tumor growth accompanied by dysfunction of immune systems through the persistent secretion of glucocorticoids. We also found that the mRNA level of perforin in spleen-derived NK cells was significantly decreased after repeated stimulation of CRH^PVN^ neurons, even though there was no change in the number of several lymphocytes from the spleen. Although it remains unclear how closely spleen-derived immune cells reflect local tumor suppression, the present findings suggest that spleen-derived NK cells could exhibit weak cytotoxicity. We next evaluated possible changes in the functions of various kinds of immune cells in the tumor microenvironment, which can directly reflect local immunity. In this study, the number of CD8^+^ T cells that infiltrated tumor tissues, which contributes to tumor suppression, was clearly and significantly decreased by repeated activation of CRH^PVN^ neurons. Since glucocorticoids have been shown to suppress metabolic pathways such as glycolysis in CD8^+^ T cells and then reduce the anti-tumor effect of CD8^+^ T cells [17], the present findings provide further evidence that endogenously released glucocorticoids by the concomitant activation of CRH^PVN^ neurons could inhibit the recruitment of CD8^+^ T cells into tumor tissues as well as the induction of dysfunctional effector lymphocytes.

It has well demonstrated that myelomonocytic lymphocytes have an adverse effect on the tumor microenvironment [18–22]. Therefore, we focused on inhibitory immune cells such as TAM and MDSC in the tumor microenvironment. In the present study, we found no change in the number of TAMs or MDSCs in tumor tissues following repeated activation of CRH^PVN^ neurons in tumor-bearing mice, whereas mRNA levels of HIF1α, GR and Tsc22d3 derived from TAMs and M-MDSCs in tumor tissues were dramatically increased by repeated activation of CRH^PVN^ neurons in these mice. HIF-1α is a key element of polarization into M2-phenotype macrophages. Both glucocorticoids and IL-10 have been shown to increase the expression of Tsc22d3, which exists selectively in M2-phenotype macrophages [23]. It has been documented that polarization of TAMs into the M2-phenotype has pro-tumor effects in the tumor microenvironment [22]. Furthermore, an important function in macrophages is efferocytosis, which facilitates immune escape in the tumor microenvironment [24–27]. It has been documented that efferocytosis is promoted in macrophages through the upregulation of Tsc22d3 [23], suggesting that Tsc22d3 could play a crucial role in promoting tumor growth. These findings imply that tumor progression due to the M2-phenotype conversion of TAMs by repeated activation of CRH^PVN^ neurons could be triggered by the upregulation of HIF1α, GR and Tsc22d3 in TAMs. Furthermore, functional changes in inhibitory immune cells such as TAMs and MDSCs as well as the depletion of CD8^+^ T cells in the tumor microenvironment could orchestrally influence the aggravation of cancer cells.

Finally, we investigated the direct influence of activating CRH^PVN^ neurons on cancer cells. It has been documented that low levels of glucocorticoids affect cell adhesion molecules, which is related to tumor suppression [28–31]. Conversely, it has been reported that glucocorticoids promote tumor metastasis and tumor growth [32, 33], suggesting that balanced glucocorticoids are important for the tumor microenvironment. In this study, we found that the activation of CRH^PVN^ neurons promoted cancer growth through the upregulation of HIF1α, Glut1, VEGFb, COX-2 and Arg1 and the downregulation of PGC1α in tumor cells, molecular events which are all directly associated with tumor progression, tumor metastasis and tumor genesis [34–37]. These results indicate that activation of the hypoxic and glycolytic pathways in cancer cells following the activation of CRH^PVN^ neurons may contribute to tumor progression.

In conclusion, we demonstrated using DREADD technology that the specific and dynamic modulation of stress-related CRH^PVN^ neurons could contribute to tumor progression via the central–peripheral-associated immune system. Although we hypothesize that the present tumor progression may mainly result from repeated activation of the HPA axis through CRH^PVN^ neurons, efferent activation of the autonomic nervous system through interactions with CRH^PVN^ neurons could be another mechanism of tumor aggravation. In addition, various humoral factors such as cytokines and lipopolysaccharides released from the tumor microenvironment could partly affect the central nervous system and result in facilitation of the afferent autonomic nervous system, leading to the promotion of tumor aggravation. Such tumor progression may be orchestrally associated with the depletion of infiltrating CD8^+^ T cells, functional conversion into M2-phenotype TAMs and changes in cancer cell properties. These findings may represent a valuable contribution toward the development of new cancer pathophysiological therapies.

## Supplementary Information

**Additional file 1: Figure S1.** Effects of the activation of CRH^PVN^ neurons on spleen-derived lymphocytes. (A, B) Representative flow cytometric dot plots (A-i, B-i) and quantitative analyses of CD4^+^ T cells (A-ii), CD8^+^ T cells (A-iii), NK cells (B-ii) and NKT cells (B-iii) derived from spleen of tumor-bearing WT/hM3Dq or CRH-Cre/hM3Dq mice. Each point represents the mean ± S.E.M. (n = 16). (C) Quantitative PCR analysis for granzyme B (C-i), perforin (C-ii), and IFN-γ (C-iii) mRNA expression in NK cells derived from spleen of WT/hM3Dq or CRH-Cre/hM3Dq mice. Each point represents the mean ± S.E.M. Unpaired *t*-test: *p < 0.05 vs. WT/hM3Dq mice (n = 16).

**Additional file 2: Table S1.** Primers sequences used for real-time qPCR.

## Data Availability

All of the data generated and analyzed in this study are included in this published article.

## Abbreviations

7-AAD: 7-Aminoactinomycin D
Arg1: Arginase 1
CNO: Clozapine *N*-oxide
COX-2: Cyclooxygenase-2
CRH: Corticotropin-releasing hormone
DMEM: Dulbecco's Modified Eagle Medium
DREADD: Designer receptors exclusively activated by designer drugs
EDTA: Ethylenediaminetetraacetic acid
FACS: Fluorescence-activated cell sorting
GAPDH: Glyceraldehyde-3-phosphate dehydrogenase
GR: Glucocorticoid receptor
Glut1: Glucose transporter 1
HIF1α: Hypoxia inducible factor 1 subunit α
hM3Dq: Gq-coupled human muscarinic M3
HPA axis: Hypothalamic–pituitary–adrenal axis
IFN-γ: Interferon-γ
IL-10: Interleukin-10
LLC: Lewis lung carcinoma
M-MDSC: Monocytic myeloid-derived suppressor cell
NK: Natural killer cell
NKT: Natural killer T cell
PBS: Phosphate-buffered saline
PD-L1: Programmed cell death 1 ligand 1
PGC1α: Peroxisome proliferator-activated receptor gamma coactivator1-α
PI: Propidium iodide
PMN-MDSC: Polymorphonuclear myeloid-derived suppressor cell
PVN: Paraventricular nucleus
TAM: Tumor-associated macrophage
TGFb1: Transforming growth factor-β1
TIL: Tumor infiltrating lymphocyte
TTDR: Tumor & Tissue Dissociation Reagent
VEGFa: Vascular endothelial growth factor a
VEGFb: Vascular endothelial growth factor b
