# Supplementary figures and images for: Elucidation of the mechanisms underlying tumor aggravation by the activation of stress-related neurons in the paraventricular nucleus of the hypothalamus
Source: Mol Brain. 2023 Feb 2;16:18. doi: 10.1186/s13041-023-01006-0 (PMC9896675; doi:10.1186/s13041-023-01006-0)

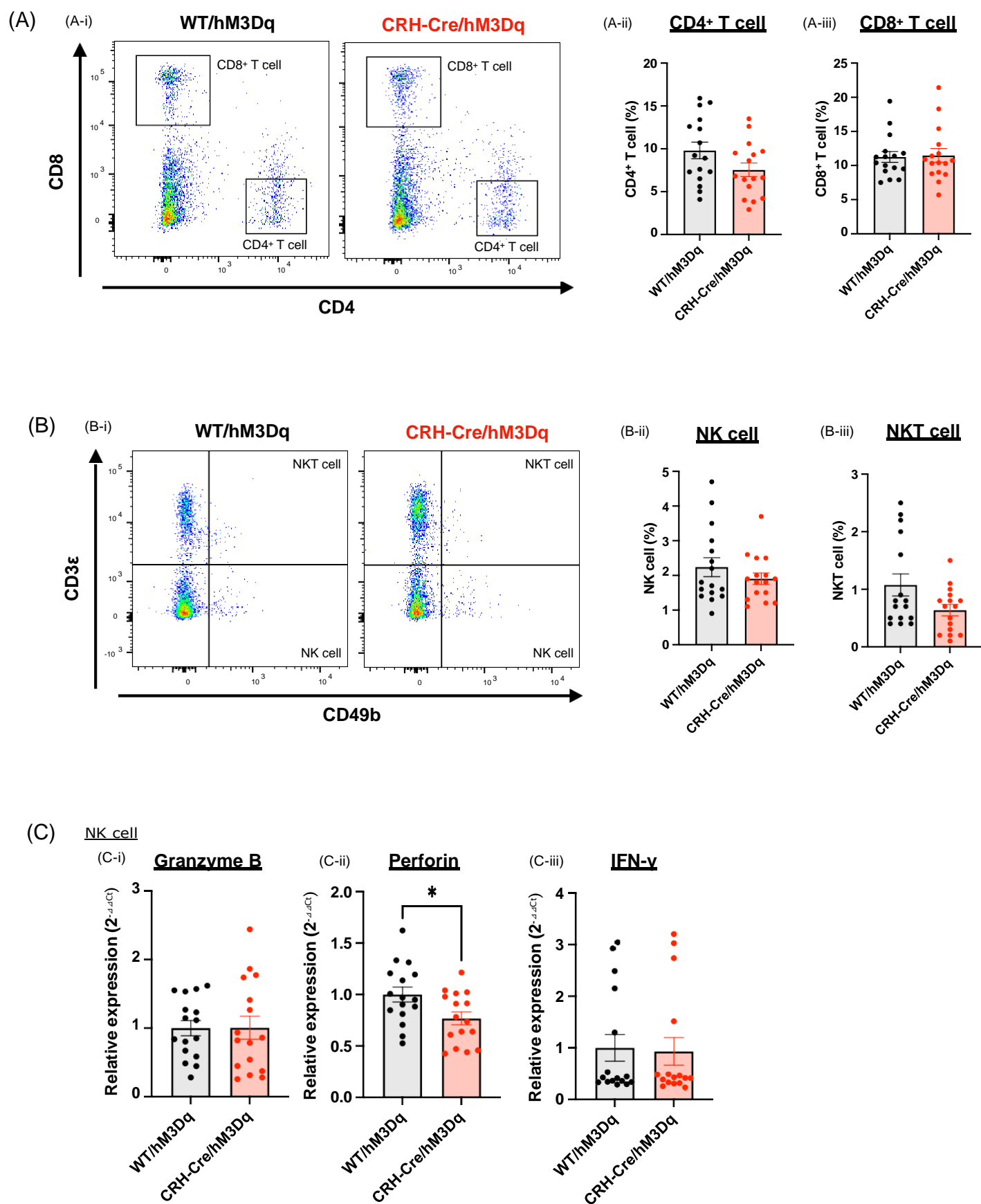

**【Figure S1】**

Supplement: Supplementary file 1 — Additional file 1: Figure S1. Effects of the activation of CRHPVN neurons on spleen-derived lymphocytes. (A, B) Representative flow cytometric dot plots (A-i, B-i) and quantitative analyses of CD4+ T cells (A-ii), CD8+ T cells (A-iii), NK cells (B-ii) and NKT cells (B-iii) derived from spleen of tumor-bearing WT/hM3Dq or CRH-Cre/hM3Dq mice. Each point represents the mean ± S.E.M. (n = 16). (C) Quantitative PCR analysis for granzyme B (C-i), perforin (C-ii), and IFN-γ (C-iii) mRNA expression in NK cells derived from spleen of WT/hM3Dq or CRH-Cre/hM3Dq mice. Each point represents the mean ± S.E.M. Unpaired t-test: *p < 0.05 vs. WT/hM3Dq mice (n = 16). [file 13041_2023_1006_MOESM1_ESM.pdf]
