# Supplementary material for: Elucidation of the mechanisms underlying tumor aggravation by the activation of stress-related neurons in the paraventricular nucleus of the hypothalamus
Source: Mol Brain. 2023 Feb 2;16:18. doi: 10.1186/s13041-023-01006-0 (PMC9896675; doi:10.1186/s13041-023-01006-0)
Supplement: Supplementary file 2 — Additional file 2: Table S1. Primers sequences used for real-time qPCR. [file 13041_2023_1006_MOESM2_ESM.docx]

**Table S1. Primers sequences used for real-time qPCR**

| **Gene** | **Forward sequence (5’→3’)** | **Reverse sequence(5’→3’)** | **Size** |
| --- | --- | --- | --- |
| GAPDH | CATGGCCTTCCGTGTTCCTA | GATGCCTGCTTCACCACCTT | 108 |
| iNOS | CCGGAGCCTTTAGACCTCAA | GCGGCTGGACTTTTCACTCT | 108 |
| NAMPT | CAGATACTGTGGCGGGAATTG | GTCTTTCCCCCAAGCCGTTA | 116 |
| TLR4 | AGGCATGGCATGGCTTACAC | TCTCCACAGCCACCAGATTCT | 120 |
| CD86 | GGAACAACTGGACTCTACGACT | TCACTGAAGTTGGCGATCACT | 145 |
| TNF | GGTGCCTATGTCTCAGCCTCTT | GCCATAGAACTGATGAGAGGGAG | 139 |
| Arg1 | GAGATTATCGGAGCGCCTTTC | CCCGTGGTCTCTCACGTCAT | 129 |
| TGFb1 | CCCCACTGATACGCCTGAGT | CAGTGAGCGCTGAATCGAAA | 105 |
| CX3CR1 | CGATTCTGCTGAGGCCTGTT | CACCAGACCGAACGTGAAGA | 97 |
| IL-10 | TTGGGTTGCCAAGCCTTATC | CACCCAGGGAATTCAAATGC | 109 |
| COX-2 | AGACAGATCATAAGCGAGGACC | GGATACACCTCTCCACCAATGA | 157 |
| Ldha | ACGCAGACAAGGAGCAGTGGAA | ATGCTCTCAGCCAAGTCTGCCA | 127 |
| HIF1α | CATCAAGTCAGCAACGTGGAA | GCACGTCATGGGTGGTTTCT | 109 |
| GR | CAAGTGATTGCCGCAGTGAA | GGCAAATGCCATGAGAAACA | 114 |
| Tsc22d3 | CCAGTGTGCTCCAGAAAGTGTAAG | AGAAGGCTCATTTGGCTCAATCTC | 188 |
| Ccr2 | GGCCACCACACCGTATGACT | TTGCCCACAAAACCAAAGATG | 123 |
| Ym-1 | TGGAATTGGTGCCCCTACAA | CAGTGGCTCCTTCATTCAGAAA | 110 |
| cMyc | GTGCTGCATGAGGAGACACC | TTGCCTCTTCTCCACAGACAC | 99 |
| Glut1 | GAATCGTCGTTGGCATCCTT | TAGCAGGGCTGGGATGAAGA | 110 |
| VEGFa | GAAGCTACTGCCGTCCGATT | GATCCGCATGATCTGCATGG | 182 |
| VEGFb | CGTTTATGCACGTGCCACAT | CACACAGCTGGGCACTAGTTG | 100 |
| PD-L1 | CGTGAGTGGGAAGAGAAGTGTC | CTACAATGAGGAACAACAGGATGG | 239 |
| Galectin-9 | TCACCTTCCAGACTCAGAACT | CTTGGACGGGTAAAGCCCAT | 188 |
| PGC1α | GAGCGCCGTGTGATTTACGT | CGGTGCATTCCTCAATTTCA | 100 |
| Perforin | CAGGTCTGGGATGCCGACTA | GCCGTGGTTTAGCTCACATGT | 105 |
| Granzyme B | CCCCAATGGGCAAATACTCA | CCCGCACATATCTGATTGGTT | 118 |
| IFN-γ | ATGAACGCTACACACTGCATC | CCATCCTTTTGCCAGTTCCTC | 182 |
